# Opportunities and challenges in implementing digital patient-centred cancer care in Europe—a qualitative study of several ESMO Designated Centres

**DOI:** 10.1016/j.esmorw.2026.100696

**Published:** 2026-03-23

**Authors:** E.D. Lundereng, A. Brkic, M. Fallon, K. Cresswell, L. Deliens, K. Pardon, K. Beernaert, A.-L. Scherrens, D. Ausen, M. Andresen, G.M. Pedersen, C. Lykke, A. Caraceni, A. Cervantes, N. Mitrea, S. Olde Damink, M.J. Hjermstad, S. Kaasa, T. Lundeby, The MyPath consortium, Kate Absolom, Morten Andresen, Marek Atter, Dag Ausen, Sara Bea, Kim Beernaert, Augusto Caraceni, Andres Cervantes, Kathrin Cresswell, Olav Dajani, Judith de Vos-Geelen, Luc Deliens, Felicity Evans, Marie Fallon, Victoria Freitas, Viviana Fusetti, Inez Gonzalez-Barrallo, Peter Hall, Marianne Jensen Hjermstad, Marisol Huerta, Kristin Solheim Hustad, An Jacobs, Stein Kaasa, Lisa Heide Koteng, Geana Paula Kurita, Henrik Larsen, Ulrik Lassen, Nicola Jane Latino, Tonje Lundeby, Elias David Lundereng, Camilla Charlotte Lykke, Giacomo Massa, Ulla Mathiesen, Nicoleta Mitrea, Daniela Mosoiu, Steven Olde Damink, Helle Pappot, Koen Pardon, Cathy Payne, Oana Predoiu, Anne-Lore Scherrens, Morena Shkodra, Per Sjøgren, Eivind Storaas, Amaia Urrizola, Peder Heyderdahl Utne, Femke Van Landschoot, Galina Velikova, Lorraine Warrington, Naomi White, Robin Williams

**Affiliations:** 1 European Palliative Care Research Centre, Department of Oncology, Oslo University Hospital and Institute of Clinical Medicine, University of Oslo, Oslo, Norway; 2 Edinburgh Cancer Research Centre, Institute of Genetics and Cancer, University of Edinburgh, Edinburgh, UK; 3 Usher Institute, University of Edinburgh, Edinburgh, UK; 4 Vrije Universiteit Brussel (VUB) & Ghent University, Department of General Practice and Chronic Care, End-of-Life Care Research Group, Brussels, Belgium; 5 DNV Imatis, Porsgrunn, Norway; 6 DNV AS, Oslo, Norway; 7 Department of Oncology and Palliative Care, North Zealand Hospital, Denmark; 8 Section of Palliative Medicine, Department of Oncology, Rigshospitalet, Copenhagen University Hospital, Copenhagen, Denmark; 9 Fondazione IRCCS Istituto Nazionale dei Tumori, Milan, Italy; 10 Dipartimento di Scienze Cliniche e di Comunità – Dipartimento di eccellenza 2023 – 2027 Università degli studi di Milano, Milan, Italy; 11 Department of Medicial Oncology INCLIVA, Biomedical Research Institute, University of Valencia, Valencia, Spain; 12 CIBERONC, Instituto Salud Carlos III, Madrid, Spain; 13 Department of Fundamental Disciplines and Clinical Prevention, University of Transilvania from Braşov, Braşov, Romania; 14 Department of Education and National Development, HOSPICE Casa Sperantei, Brasov, Romania; 15 Department of Surgery, Maastricht University Medical Centre, Maastricht, The Netherlands; 16 NUTRIM Institute of Nutrition and Translational Research in Metabolism, Maastricht University, Maastricht, The Netherlands; 17 Department of General, Visceral, Vascular and Transplant Surgery, University Hospital Essen, Essen, Germany; 18 MATRIX, Norwegian Centre for Clinical Cancer Research, Oslo, Norway

**Keywords:** digital health, patient-reported outcome measures, integrated care, person-centred care, continuity of care, health services research

## Abstract

**Background:**

Patient-centred care (PCC) improves patient outcomes in cancer care, such as survival and quality of life, yet it remains inconsistently implemented across Europe due to system- and provider-level barriers that favour disease-centred models. Guided by expert perspectives from the ESMO Designated Centres (DCs), the European Union-funded MyPath project is developing a digital solution incorporating patient-reported outcome measures to operationalise PCC across diverse European contexts. This study aims to explore challenges and opportunities for implementing digital solutions to enhance PCC in oncology, ensuring relevance and scalability across Europe using insights from ESMO DCs.

**Materials and Methods:**

A qualitative study was conducted through three semistructured focus groups with 21 clinicians and leaders from 14 ESMO DCs across 14 European countries. Qualitative analysis, guided by the Technology, People, Organisations, and Macroenvironment (TPOM) framework, identified multilevel implementation factors.

**Results:**

The analysis identified three main themes: (i) Challenges in adapting a digital PCC solution across diverse health care settings, where heterogeneity in health care organisation, resources, and political systems challenges universal implementation; (ii) The complexity of providing PCC in digital formats, highlighting the tension between standardised and flexible care; and (iii) The need for training and cultural shifts to support successful implementation, describing the need to adjust clinical behaviours and workflows to foster PCC.

**Conclusions:**

Effective digital PCC depends not only on technical integration and clinical training but also on adaptability to different care settings, infrastructures, and professional cultures. This study highlights the conditions for scalable implementation of digital PCC across Europe, offering practical implications for health system leaders and policymakers.

## Highlights

• MyPath aims to develop and implement a digital solution for patient-centred cancer care using patient-reported outcomes.
• Participants from ESMO DCs contributed expert opinions on the development and implementation of MyPath.
• Digital tools offer promising opportunities to integrate a systematic and evidence-based patient-centred approach.
• Scaling a digital solution for implementation across Europe requires adaptability and flexibility across European contexts.
• Organisational and professional cultures that favour a disease-centred approach over PCC must be overcome.

## Introduction

Advancements in cancer detection and treatment enable more people with cancer to live longer. However, as patients live longer while navigating novel treatments and their sometimes complex adverse effects, the burden of physical, psychological, emotional, spiritual, and social challenges related to the disease and its treatment intensifies.^1^^,^^2^ Consequently, a purely tumour-centred care approach, primarily focused on the disease itself, is increasingly inadequate.^2^ In response, patient-centred care (PCC), which emphasises patients’ preferences, values, and psychosocial needs, has emerged as an increasingly recognised model in oncology.^3^^,^^4^ Integrating PCC throughout the cancer disease trajectory reduces symptom burden and improves quality of life, treatment adherence, and even survival outcomes.^5^, ^6^, ^7^, ^8^

Recognising the value of PCC, major organisations such as the World Health Organisation (WHO), the ESMO, the American Society of Clinical Oncology (ASCO), and the European Association for Palliative Care (EAPC) advocate for PCC implementation.^9^, ^10^, ^11^, ^12^ In Europe, initiatives such as the Joint Action on Networks of Expertise on Cancer (JANE-2) and the European Union (EU) Mission on Cancer emphasise the importance of addressing patient experiences across the care continuum.^13^, ^14^, ^15^ However, barriers related to institutional culture, workflow and resource limitations, and a preference among health care providers (HCPs) to focus on tumour-centred care limit systemic adoption.^2^^,^^16^, ^17^, ^18^ Besides, PCC remains difficult to translate into real-world clinical practice because of its philosophical and vague definitions.^19^

In response to this, patient-reported outcome measures (PROMs) have emerged as a promising way to operationalise PCC in oncology. By capturing patients’ symptoms, functioning, and quality of life directly from their perspective, PROMs provide a framework that enables more patient-centred consultations.^20^^,^^21^ Digital solutions incorporating PROMs extend this potential by enhancing communication and access, supporting individualised, data-driven PCC.^22^, ^23^, ^24^, ^25^, ^26^, ^27^, ^28^ However, widespread adoption remains challenging due to issues of scalability, usability, data security, and challenges with adapting existing routines.^25^^,^^29^ Moreover, only a few digital oncology initiatives to date have focused specifically on advancing PCC.^28^

To address this gap, the EU-funded MyPath project aims to develop and implement a digital solution for integrated PCC. The system incorporates digital PROMs that link to evidence-based guidelines, facilitating structured, PCC pathways for symptom management. Guided by implementation science methodologies, stakeholder engagement, and mapping of clinical workflows is conducted to ensure applicability and scalability across diverse pan-European settings, based on user needs.^30^, ^31^, ^32^, ^33^

Successful implementation depends on staff engagement and leadership support. Management plays a pivotal role in shaping institutional priorities, allocating resources, and driving change, yet little is known about their views on PCC and digital innovation in oncology across different health care systems in Europe. This study aimed to generate insights that could inform the design and implementation strategies of MyPath from the perspective of HCPs and managers at ESMO Designated Centres (DCs), institutions recognised for integrated oncology and palliative care.^34^ Specifically, this study is guided by the following research questions: (i) how is PCC currently understood, practised, and experienced by HCPs and managers at ESMO DCs, and (ii) what challenges and opportunities influence the integration of PCC and digital tools such as digital PROMs into oncology workflows?

## Materials and methods

MyPath, a multinational, 5-year EU-funded implementation science project, aims to develop and implement a digital solution for PCC in nine cancer centres across Europe. The project is guided by a systematic, theory-based mixed-methods approach, combining qualitative and quantitative data to iteratively guide implementation through improving and adapting the solution to diverse organisational contexts.^35^

The Technology, People, Organisations, and Macroenvironment (TPOM) framework is the evaluative framework, providing an evidence-based lens on digital health adoption.^36^ To ensure relevance and scalability across European health care settings, ESMO DCs were engaged for their recognised expertise in integrated oncology and palliative care. This study reports findings from a formative-stage workshop, which focused on conceptual aspects of the solution, including PCC delivery, the use of PROMs, and the potential for digital tools to support PCC. Findings from this study will inform the ongoing design and implementation strategies of MyPath, grounding the solution in expert perspectives across Europe.

### Study design

This study employed a qualitative, interpretive design grounded in Interpretive Description (ID),^37^ a methodology that emphasises exploration of participants’ experiences in clinical contexts to generate practical, contextually relevant knowledge for guiding development and implementation strategies in health care. Semistructured focus group discussions with staff and leadership at ESMO DCs were conducted to capture perspectives on complex, real-world needs for digitally enabled PCC. The study followed the Consolidated Criteria for Reporting Qualitative Research (COREQ) checklist^38^ to ensure rigour and transparency in reporting.

### Participants and recruitment

Participants were purposively sampled from ESMO DCs across Europe to ensure broad geographic and professional representation. Representatives from 20 DCs were invited, and 21 participants from 14 DCs accepted, representing 14 countries (Austria, Czech Republic, Denmark, Finland, France, Germany, Hungary, The Netherlands, Poland, Portugal, Romania, Slovenia, Spain, and The United Kingdom). Before the workshop, participants attended a preliminary digital meeting to clarify project aims. Participant characteristics are summarised in Table 1.

**Table 1:** Demographics of the study participants

Participants’ characteristics	Values (*n* = 21), *n* (%)
Sex	
Female	13 (62)
Male	8 (38)
Position	
Clinical	12 (57)
Management	7 (33)
Academic	2 (10)
Professional roles	
Palliative care	8 (38)
Oncology (medical)	6 (29)
Radiation oncology	4 (19)
Nursing	2 (10)
Academic roles	1 (4)

### Data collection

Data were collected before and during an in-person workshop in Brussels in September 2023. Before the workshop, participants completed a brief online survey with mixed quantitative and open-ended questions on PROM collection, digital tool use, workflow impact, and institutional adaptability, providing contextual data for each centre reported in the ‘Results’ section (Table 4).

Participants were divided into three groups (A, B, and C), each taking part in two semistructured group discussions (sessions 1 and 2) facilitated by six experienced researchers in pairs using preprepared interview guides (Tables 2 and 3). In session 1, groups discussed conceptual aspects of PCC implementation (facilitators and barriers, current practices, digital system use, and workplace culture). This was followed by a plenary session where all participants reviewed digital solution mock-ups and proposed workflows. Session 2 then focused on technical and implementation factors, such as workflow integration, digital adoption, and anticipated impact on patients and HCPs.

**Table 2:** Condensed interview guide for session 1: the concept of patient-centred care

Topic	Questions
Current PCC practices in oncology care	How do you incorporate PCC in your work with cancer patients?
	How do you address symptoms such as pain or depression?
	What facilitates or hinders your ability to provide PCC?
	How would you assess the current level of PCC? How could it be improved?
Use of digital systems	How do you currently use digital tools (e.g. EHRs) to support PCC?
	How are they used within teams and with patients?
	What are the benefits and challenges?
	How receptive is your workplace to adopting new digital systems?
Digital PROMs and care pathways	What are your thoughts on using digital PROMs to systematically measure patient outcomes?
	How could such data be integrated into consultations and workflows?
	What would encourage or prevent you from using this tool?
	What resources (e.g. training, leadership support) would be needed for successful implementation?
Closing	Any final thoughts, questions, or additional input?

EHR, electronic health record; PCC, patient-centred care; PROM, patient-reported outcome measure.

**Table 3:** Condensed interview guide for session 2: technical aspects and MyPath digital solution

Topic	Questions
Prototype evaluation	What are your overall impressions of the MyPath prototype?
	Are any functions/domains missing or unnecessary?
	Thoughts on the graphical user interface? Likes/dislikes?
Practical application	How could this tool benefit your work and cancer treatment?
	What advantages and challenges do you foresee?
Closing	Summary of key points—do you agree? Any final comments or questions?

All recorded sessions (*n* = 7) were transcribed verbatim. Session 1 averaged 54 min (range 52-59 min), followed by the plenary session, which lasted for 48 min. Session 2 averaged 78 min (range 73-85 min).

### Data analysis

Analysis was guided by ID, emphasising the identification of insights and patterns relevant to clinical practice and implementation. Transcripts were read in full by two researchers (EDL and AB) to gain familiarity and develop initial impressions. Data were organised using a deductive–inductive approach, with deductive coding guided by the TPOM framework, and inductive coding to capture additional concepts and patterns not covered by TPOM. Codes were reviewed and grouped into categories across TPOM themes and subthemes, reflecting key implementation considerations, practical barriers and facilitators, and workflow or cultural factors. NVivo (Lumivero, Burlington, MA) was used to facilitate coding.

**Figure 1:**
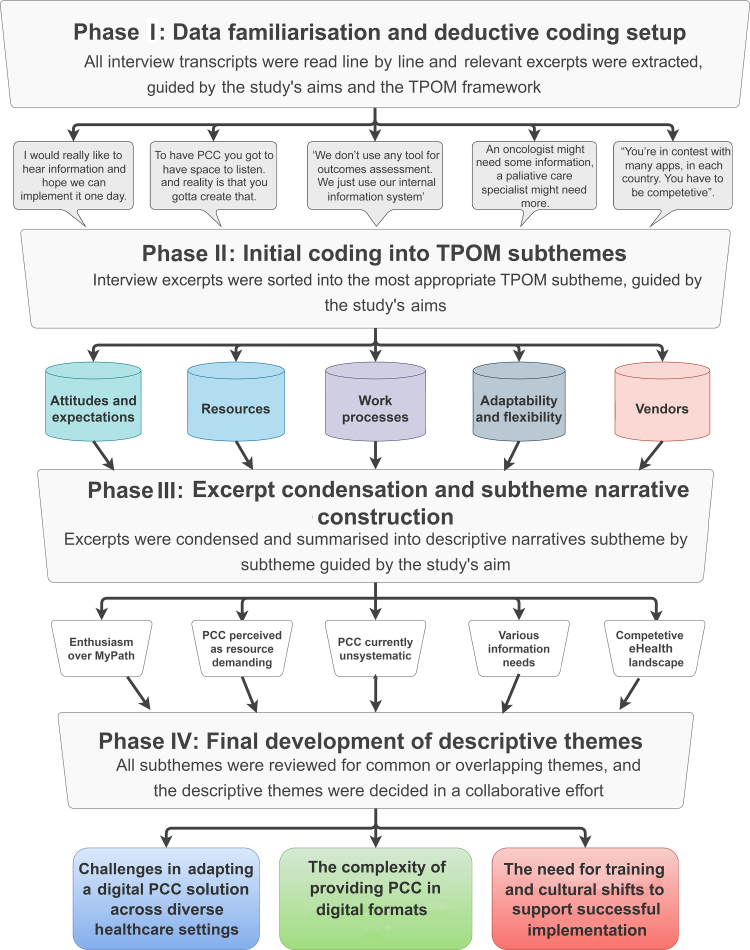
**Illustration of the analysis process from source material to descriptive themes.** PCC, patient-centred care; TPOM, technology, people, organisations, and macroenvironment.

To enhance rigour, coding and category development were conducted collaboratively, with regular coauthor discussions to reconcile differences and refine interpretations. Researchers remained reflexive throughout, considering how their professional backgrounds and experiences with PCC and digital tools may have influenced the analysis. This approach allowed the identification of clinically meaningful patterns and actionable insights across participating centres. The analytic process is illustrated in Figure 1.

### Ethics approval and consent to participate

The study was reviewed by the Regional Committees for Medical and Health Research (REK) in Norway (application number 633827) and deemed not to require formal ethical approval, as it did not fall under the definition of health research under Norwegian law. All data collection and management followed the Oslo University Hospital procedures for secure and responsible handling of research data. Participants received written and verbal information about the study, provided informed consent, and were assured that participation was voluntary and that they could withdraw at any time.

## Results

### Survey results

Of the 21 participants, 19 responded to the survey, representing all 14 participating centres, providing contextual background on each participating centre. Responses were divided into three main themes: current practice and use of PROMs; digital PROMs integration and challenges; and future directions for implementation. Survey responses are detailed in Table 4.

**Table 4:** Condensed survey findings

Question	Response
1. How do you collect patient-reported outcomes at your centre?	Digitally (*n* = 12) Paper record (*n* = 6) Both (*n* = 1)
2. Which instruments do you use to collect PROMs?	Validated instruments mentioned are iPOS, ESAS, EORTC (QLQ-C30, QLQ-C15-PAL), HADS, Distress Thermometer, Melzack–McGill, MMSE, FICA, VAS/NRS, KPS, and ECOG.
3. Please describe how these PROMs are used at your centre	Research only (*n* = 3) Clinical use (*n* = 10) Both research and clinical use (*n* = 6)
4. If you use digital PROMs at your centre, in your opinion, has the integration of digital software affected patient care and workflow?	Yes (*n* = 9) No (*n* = 2) Not applicable (*n* = 8)
5. If you have used digital PROMs, what implementation challenges have you faced, and how did you address them?	Technical integration issues (*n* = 6) Patient compliance and literacy (*n* = 3) Staff adoption and training (*n* = 4) No response (*n* = 6)
6. Have you been involved in implementing digital decision support tools in patient-centred care previously?	Yes (*n* = 7) No (*n* = 10) No response (*n* = 2)
7. If yes (*n* = 7), please describe your experience below	Research participation (*n* = 2) Clinical implementation (*n* = 3) Pilot projects (*n* = 2)
8. In MyPath, digital PROMs will be linked to computer-based decision support: what are your thoughts about that?	Positive (*n* = 15) Neutral (*n* = 2) Sceptical (*n* = 1) No response (*n* = 1)
9. In MyPath, digital PROMs will be linked to computer-based decision support: have you had similar experiences?	Yes (*n* = 6) No (*n* = 11) No response (*n* = 2)
10. Would you describe your centre as adaptable to change?	Yes (*n* = 19) No (*n* = 0)
11. What do you believe is necessary to promote positive change?	Leadership support (*n* = 5) Training and education (*n* = 6) Clear implementation plans (*n* = 4) Technical support and funding (*n* = 3) No response (*n* = 1)

Distress Thermometer, a single item numeric screening tool for psychological distress; ECOG, Eastern Cooperative Oncology Group Performance Status; EORTC, European Organisation for Research and Treatment of Cancer; ESAS, Edmonton Symptom Assessment System; FICA, Faith, Importance/Influence, Community, Address (spiritual assessment tool); HADS, Hospital Anxiety and Depression Scale; iPOS, Integrated Palliative Care Outcome Scale; KPS, Karnofsky Performance Status; Melzack–McGill, McGill Pain Questionnaire; MMSE, Mini-Mental State Examination; NRS, Numeric Rating Scale; PROM, patient-reported outcome measure; QLQ-C15-PAL, Quality of Life Questionnaire-Core 15 for Palliative Care; QLQ-C30, Quality of Life Questionnaire-Core 30; VAS, Visual Analog Scale.

#### Current practice and use of PROMs

Collection of PROMs across the participating centres was primarily digital (*n* = 12), with six centres using paper-based PROMs. The primary use for PROMs was clinical (*n* = 10); however, six centres utilised them for both clinical and research purposes. Only three centres used them solely for research.

#### Digital PROM integration and challenges

Among the 12 centres using digital PROMs, 9 felt that the tools had positively impacted patient care and workflows. Despite the perceived benefits, the most frequently reported challenges were technical integration (*n* = 6), issues related to staff adoption and training (*n* = 4), and challenges with patient compliance or digital literacy (*n* = 3). These findings suggest that successful adoption requires addressing both technological and human factors.

#### Future directions for implementation

Linking digital PROMs to evidence-based guidelines was viewed positively by the majority of respondents (*n* = 15). In addition, all respondents (*n* = 19) described their centre as adaptable to change, highlighting a key strength for future implementation efforts. The most commonly cited factors to successful implementation were training and education (*n* = 6), leadership support (*n* = 5), clear implementation plans (*n* = 4), and adequate technical support and funding (*n* = 3).

### Primary results

The qualitative analysis of the discussions identified three main themes: (i) challenges in adapting a digital PCC solution across diverse health care settings; (ii) the complexity of providing PCC in digital formats, and (iii) the need for training and cultural shifts to support successful implementation. These results build on the survey findings, providing additional context on current practices, the use of PROMs and digital tools, and considerations for future implementation.

#### Challenges in adapting a digital PCC solution across diverse health care settings

This theme emphasises that implementing a digital PCC solution across diverse European oncology settings is challenging due to variations in health care organisation, resources, and political systems. Central to the theme was the need for flexibility to accommodate variations in health care organisation, as well as cultural and linguistic differences requiring modular and adaptable tools, as one oncologist explained:

“The most important thing is that tools need to be adaptable to our situations. Hospitals should be able to use the modules they need. Otherwise, initial enthusiasm goes away when technical challenges arise. A flexible approach ensures the tool remains useful”—Oncologist 1, group B

In addition, resource disparities across Europe were considered a major barrier. PCC was seen as resource demanding due to the required collection and assessments of PROMs, initiation of treatment, and structured follow-up. Workforce shortages in allied health, mental health, and palliative care were perceived as limiting factors, especially in smaller or economically constrained hospitals or countries. Larger centres with multidisciplinary teams were perceived as better positioned to implement MyPath, as illustrated by an oncologist:

“We’re lucky to have many resources and a well-structured team, which makes implementation easier. But I know this can be very different in general hospitals or smaller centres, where supportive and palliative care resources may be lacking.”—Oncologist 3, group C

However, larger centres also faced challenges as multiple teams across different sites complicated coordination. This fragmentation made it difficult to involve everyone responsible for symptom management and to integrate new tools, as one oncologist explained:

“At our large hospital with multiple sites, there’s no standardised pathway to integrate palliative care into oncology. Many teams across wards provide specialist and supportive care, which makes coordination complex.”—Palliative physician 3, group C

Moreover, participants emphasised that successful implementation would require overcoming substantial organisational challenges. Hospitals’ willingness to adopt new tools was influenced by national funding priorities and political incentives, which varied across countries. The competitive electronic health (eHealth) landscape also meant MyPath would need to clearly demonstrate its added value. Strong endorsement from important organisations was seen as a facilitating factor, as illustrated by an oncologist:

“There is a lot of competition, but an EU-funded and ESMO-recommended solution that follows existing guidelines and lets you check whether the guidelines were followed would be very useful at the European level.”—Oncologist 4, group A

#### The complexity of providing PCC in digital formats

The second theme highlighted the tension between standardised and flexible care. While participants recognised that standardising care using digital tools and PROMs could improve the management of physical symptoms, they were concerned that these tools might fail to capture the psychosocial aspects of care, which they perceived as requiring a more nuanced approach. As one oncologist explained:

“We often focus on pain, nausea, and vomiting, but it’s not always easy to identify the main issue. For my patients, talking openly is important, but sometimes it’s hard to see what really matters. To address symptoms properly, we need to broaden the scope to better understand the real problem.”—Oncologist 1, group C

Furthermore, while PROMs provided numerical overviews of symptom severity, their subjective interpretation often led to inconsistent actions. Integrating evidence-based guidelines to support decision making was seen to enable more consistent symptom management, particularly in areas like neuropathic pain, where practices differed. As one palliative physician explained:

“MyPath would be really great if it continuously recorded patient needs with links to the guidelines. PROMs help identify symptoms, but different doctors prescribe different treatments, not always following the guidelines”—Palliative physician 3, group C

Participants also expressed concerns about the rigidity of structured care pathways with predefined management guidelines. Participants described that care decisions in PCC settings require a deep understanding of individual preferences and strong collaboration between patients, caregivers, and clinicians. While standardisation was viewed as feasible for other aspects of medicine, a palliative care setting required more nuances, as illustrated by a participant:

“In some areas of medicine, treatment is straightforward. But in palliative care, drug choices are complex, influenced by side effects, compliance, timing, and beliefs. Even with clear symptoms, guidelines offer multiple options. A one-size-fits-all approach doesn’t work here.”—Palliative physician 1, group A

Participants agreed that a digitally enabled PCC would require extensive data collection, posing challenges across care contexts. They felt that palliative care teams, with their natural alignment to the holistic nature of PCC, would be more likely to adopt MyPath. By contrast, oncology settings, which often focus on treatment-related side-effects, might find the adoption of comprehensive psychosocial and symptom data more difficult to integrate into standard consultations. As ensuring oncologist buy-in was considered essential, some suggested a simplified version tailored to their needs as a starting point:

“As an oncologist, it’s important to focus on quick and important information. We should prioritise important symptoms at the first step. Too many details, and we won’t know what to deal with, and it is difficult to identify the main issue”—Oncologist 1, group C

#### The need for training and cultural shifts to support successful implementation

The third theme describes the need to adjust clinical behaviours to foster PCC. Participants noted that oncology practice often remains tumour centred, with psychosocial concerns frequently overlooked unless prompted by the patient. While digital tools can help patients express these experiences, participants emphasised that MyPath must also foster clinical accountability, ensuring that PCC is treated as an essential requirement of the consultation rather than an optional addition. As one oncologist observed:

“Many oncologists are medically oriented, not patient-oriented. They ask ‘How are you?’, the patient says “I’m fine,” and they proceed with treatment. That’s not patient-centred. Can MyPath change this? Maybe, if it provides clear direction and makes a patient-centred approach the responsible choice.”—Oncologist 2, group A

Participants also emphasised how clinical cultures shaped patient expectations, with patients accustomed to primarily reporting physical symptoms and side-effects, while psychosocial or emotional concerns were deprioritised. One participant reflected on how their own consultation style was shaped by clinical pressures and limited space for PCC, creating a narrow focus:

“As a medical oncologist seeing 20-40 patients daily, I train them to report specific issues like neuropathy or diarrhoea. But beyond those specific concerns, many other aspects of their experience are not discussed.”—Oncologist 2, group C

Despite the promise of digital tools, participants emphasised that digital innovations often faced resistance rooted in perceptions of digital systems being threats to clinical autonomy, discomfort with new technologies, lack of integration with existing systems or concerns about increased workload. Participants described senior HCPs as more reluctant to adopt digital tools, as one nurse described from past experiences with implementing a digital solution:

“At first, it was just about figuring out how to use it, and it took a lot of time, especially for the older staff who found changing systems difficult. But now we have new staff, and for them, it’s not a problem anymore. So yes, things are changing, and we are seeing the possibilities of the solution. But it takes time.”—Nurse 1, group 2

Participants with prior experience using electronic PROMs emphasised that, once initial apprehension was overcome, implementation led to improvements in both workload and care quality. However, participants emphasised that successful adoption required training that went beyond technical instruction. Instead, training should focus on how to use the information meaningfully in clinical consultations, moving beyond simply collecting PROMs to using them to reshape the scope of clinical interactions, as one participant noted:

“The problem isn’t collecting outcomes, we have all kinds of tools for that. The biggest problem is getting clinicians to actually use them with the patients during consultations, to support shared decision-making”—Palliative care physician 4, group C

## Discussion

This study explored current PCC practices, the use of digital solutions, and the potential role of MyPath in supporting PCC in oncology from the perspectives of ESMO DCs. The results highlight the interplay between technology, clinical workflows, and professional culture. Key considerations for successful implementation include the need for adaptable digital tools, the balance between standardisation and clinical flexibility, and the importance of training and cultural change to support meaningful use of PROMs. These considerations will shape the development and implementation of MyPath in European oncology settings.

A key theme was the need for adaptability, interoperability, and integration across Europe’s diverse health care contexts. Interestingly, while participants perceived their centres as adaptable, discussions focused on the need to modify digital systems to fit existing practices. This reflects a broader eHealth challenge where institutions are hesitant to adopt solutions that require workflow changes, due to the time and effort involved.^39^, ^40^, ^41^ Consequently, many prioritise adapting digital tools to fit current processes rather than undertaking the necessary structural changes for full integration.^28^^,^^41^, ^42^, ^43^ These barriers highlight that, while technical adaptability and interoperability are prerequisites, successful implementation requires change management that evolves clinical workflows to better leverage digital capabilities.^28^^,^^44^

Another key concern was that MyPath could overwhelm already burdened health care services, reflecting research showing that electronic PROMs may initially disrupt workflows and add administrative burdens.^41^^,^^42^ However, when successfully implemented, these solutions can enhance efficiency, job satisfaction, and health care utilisation through optimising overall clinical capacity.^45^^,^^46^ These findings highlight the need for implementation strategies that enable HCPs to experience first-hand the potential long-term benefits of adopting digital PCC tools.

Participants highlighted the importance and challenge of fostering acceptance among oncologists. As oncologists’ buy-in was seen as essential, aligning MyPath with oncologists’ needs, through emphasising treatment-related symptoms, was suggested to encourage adoption. However, while HCP buy-in is a key driver of successful implementation,^47^^,^^48^ over-tailoring the solution to meet oncologists’ needs risks undermining PCC principles. Defining a minimum viable solution that enhances PCC while still promoting early adoption may help mitigate the risk of reducing MyPath to a provider-centred tool.

While technical training is essential in digital health implementation,^41^^,^^44^ participants in our study highlighted the need for training in applying PROMs in clinical settings. This is supported by research showing that HCPs often struggle to apply PROMs effectively with patients within time-pressured clinical realities.^49^^,^^50^ Rather than treating PROMs as fixed values, they should serve as a starting point for dialogue about patient needs.^43^^,^^51^^,^^52^ This underscores the need for digital tools to facilitate meaningful, patient-centred consultations rather than reducing PCC to a checklist. Addressing this requires guidance on integrating PROMs into consultations, fostering a culture that prioritises PCC.

A key finding of this study is the tension between standardisation and clinical autonomy. While evidence suggests that standardisation can improve the consistency and quality of care,^8^^,^^53^ participants expressed concerns that standardisation could reduce their ability to provide individualised PCC. This is consistent with research showing that HCPs’ resistance to digital tools is often rooted in a perceived threat to professional autonomy and clinical judgement.^54^^,^^55^ Positioning digital tools as decision support, where outputs complement rather than replace clinical evaluation, can help balance the need for standardisation with the flexibility required for nuanced PCC.

Ultimately, while MyPath cannot address all systemic challenges to PCC integration, it aims to support initiatives across Europe through alignment with and support of the objectives of the EU’s Mission Cancer^15^ and contributions to the palliative care work package of JANE-2.^13^^,^^14^ Achieving meaningful progress in cancer care will require continuous, coordinated, and collaborative efforts within and between pan-European initiatives. MyPath can hopefully provide an actionable and clinically relevant framework to integrate PCC using digital tools across Europe.

### Strengths and limitations

While this qualitative study provides in-depth insights into the implementation of MyPath across Europe, some limitations should be noted. The sample, purposefully drawn from ESMO DCs for their expertise in integrative care, is not representative of average European cancer institutions. The relatively high uptake of digital PROMs and other digital tools reported in this study likely reflects the expertise and resources of participating ESMO DCs. Therefore, experiences may differ in contexts with fewer resources, necessitating engagement with other centres to inform the scalability of MyPath across Europe. The simultaneous 1-day data collection limited reflexivity and the ability to build on insights between groups. However, structured interview guides and theory-driven analysis ensured consistency in data collection and analysis. Data saturation was not assessed, and repeated interviews might have provided greater depth. Repeated involvement of ESMO DCs will track how perceptions evolve during implementation, and how digital solutions can support cultural shifts towards PCC in oncology.

### Conclusions

This study highlights how digital tools like MyPath can be implemented to support PCC in oncology across Europe by standardising symptom tracking and management. The results highlight the technical requirements for adaptability, interoperability, and digital integration for successful scalable implementation across Europe. However, successful adoption depends not only on technology but also on embedding PCC into organisational culture, providing technical and clinical training, and securing leadership support. The findings underscore the interplay between technological, professional, and organisational factors, offering actionable guidance for designing scalable, context-sensitive digital PCC solutions across diverse European settings.
