# Supplementary material for: Opportunities and challenges in implementing digital patient-centred cancer care in Europe—a qualitative study of several ESMO Designated Centres
Source: ESMO Real World Data Digit Oncol. 2026 Mar 23;12:100696. doi: 10.1016/j.esmorw.2026.100696 (PMC13049654; doi:10.1016/j.esmorw.2026.100696)
Supplement: Supplementary Material S1 [file mmc1.pdf]

MyPath Consortium

Kate Absolom[1,2], Morten Andresen[3], Marek Atter[4], Dag Ausen[3], Sara Bea[4], Kim Beernaert[5], Augusto Caraceni[6-7], Andres Cervantes[8-9], Kathrin Cresswell[10], Olav Dajani[11], Judith de Vos-Geelen[12], Luc Deliens[5], Felicity Evans[13], Marie Fallon[4], Victoria Freitas[14], Viviana Fusetti[6], Inez Gonzalez-Barrallo[14], Peter Hall[4], Marianne Jensen Hjermstad[11], Marisol Huerta[14], Kristin Solheim Hustad[11], An Jacobs[15], Stein Kaasa[11], Lisa Heide Koteng[11], Geana Paula Kurita[16,17], Henrik Larsen[18], Ulrik Lassen[17,19], Nicola Jane Latino[13], Tonje Lundeby[11], Elias David Lundereng[11], Camilla Charlotte Lykke[18,20], Giacomo Massa[6,7], Ulla Mathiesen[18], Nicoleta Mitrea[21,22], Daniela Mosoiu[22,23], Steven Olde Damink[24-26], Helle Pappot[17-19], Koen Pardon[5], Cathy Payne[27], Oana Predoiu[22], Anne-Lore Scherrens[5], Morena Shkodra[11], Per Sjøgren[18], Eivind Storaas[11], Amaia Urrizola[11], Peder Heyderdahl Utne[11], Femke Van Landschoot[5], Galina Velikova[1-2], Lorraine Warrington[2], Naomi White[4], Robin Williams[28]

[1] Leeds Cancer Centre, St James's University Hospital, Leeds, UK

[2] Leeds Institute of Medical Research at St James's, University of Leeds, Leeds, UK

[3] DNV Imatis AS, Porsgrunn, Norway

[4] Institute of Genetics and Cancer, University of Edinburgh, Edinburgh, UK

[5] Vrije Universiteit Brussel (VUB) & Ghent University, Department of General Practice and Chronic Care, End-of-Life Care Research Group, Brussels, Belgium

[6] Fondazione IRCCS Istituto Nazionale dei Tumori, Milan, Italy

[7] Dipartimento di Scienze Cliniche e di Comunità – Dipartimento di eccellenza 2023 – 2027 Università degli studi di Milano, Milan, Italy

[8] Department of Medical Oncology INCLIVA, Biomedical Research Institute, University of Valencia, Valencia, Spain

[9] CIBERONC, Instituto Salud Carlos III, Madrid, Spain

[10] Usher Institute, University of Edinburgh, Edinburgh, United Kingdom

[11] European Palliative Care Research Centre (PRC), Department of Oncology, Oslo University Hospital, and Institute of Clinical Medicine, University of Oslo, Oslo, Norway

[12] Department of Internal Medicine, Division of Medical Oncology, GROW - Research Institute for Oncology & Reproduction, Maastricht University Medical Center+, Maastricht, the Netherlands

[13] Department of Scientific & Medical Affairs, European Society for Medical Oncology (ESMO), Lugano, Switzerland

[14] Department of Medical Oncology INCLIVA, Biomedical Research Institute, Valencia, Spain

[15] Vrije Universiteit Brussel (VUB), Department of Media and Communication Studies, Imec-SMIT Research Group, Brussels, Belgium

[16] Rigshospitalet Copenhagen University Hospital, Department of Oncology and Department of Anaesthesiology, Pain and Respiratory Support, Copenhaguen, Denmark

[17] University of Copenhagen, Department of Clinical Medicine, Copenhaguen, Denmark

[18] Rigshospitalet Copenhagen University Hospital, Department of Oncology, Section of Palliative Medicine, Copenhaguen, Denmark

[19] Rigshospitalet Copenhagen University Hospital, Department of Oncology, Copenhaguen, Denmark

[20] Department of Oncology and Palliative Care, North Zealand Hospital, Denmark

[21] Department of Fundamental Disciplines and Clinical Prevention, Faculty of Medicine, University of Transilvania from Brasov, Romania

[22] Department of Education and National Development, HOSPICE Casa Sperantei, Brasov, Romania

[23] Department of Medical and Surgical Specialties, Faculty of Medicine, University of Transilvania from Brasov, Romania

[24] Department of Surgery, Maastricht University Medical Centre, Maastricht, The Netherlands

[25] NUTRIM Institute of Nutrition and Translational Research in Metabolism, Maastricht University, Maastricht, The Netherlands

[26] Department of General, Visceral, Vascular and Transplant Surgery, University Hospital Essen, Essen, Germany

[27] European Association for Palliative Care, Belgium

[28] Institute for the Study of Science, Technology and Innovation, University of Edinburgh, Edinburgh, United Kingdom
